# A cross-country comparison of Covid-19 containment measures and their effects on the epidemic curves

**DOI:** 10.1186/s12889-022-14088-7

**Published:** 2022-09-17

**Authors:** Fidelia Cascini, Giovanna Failla, Cecilia Gobbi, Elena Pallini, Jin Hui, Wang Luxi, Leonardo Villani, Wilm Quentin, Stefania Boccia, Walter Ricciardi

**Affiliations:** 1 Section of Hygiene, University Department of Life Sciences and Public Health, Università Cattolica del Sacro Cuore, 00168 Roma, Italy; 2 Data Science & Advanced Analytics, IQVIA, 20124 Milan, Italy; 3 Integrated Analytics, IQVIA, 20124 Milan, Italy; 4 Data Science & Advanced Analytics, IQVIA, Bejing, 100006 China; 5 Sales Effectiveness, Marketing Commercial Excellence, Novo Nordisk, Beijing, 100102 China; 6 Department of Health Care Management, Technische Universität Berlin, 10623 Berlin, Germany; 7 European Observatory on Health Systems and Policies, 1060 Brussels, Belgium; 8 Department of Woman and Child Health and Public Health - Public Health Area, Fondazione Policlinico Universitario A. Gemelli IRCCS, Roma, Italy

**Keywords:** Covid-19, Pandemic, Restrictions, Containment measures, Health policies

## Abstract

**Background:**

European countries are still searching to eliminate or contain the Covid-19 pandemic. A variety of approaches have achieved different levels of success in limiting the spread of the disease early and preventing avoidable deaths. Governmental policy responses may explain these differences and this study aims to describe evidence about the effectiveness of containment measures throughout the course of the pandemic in five European countries (France, Germany, Italy, Spain and the UK).

**Methods:**

The research approach adopted consisted of three steps: 1) Build a Containment Index (C.I.) that considers nine parameters to make an assessment on the strength of measures; 2) Develop dynamic epidemiological models for forecasting purposes; 3) Predict case numbers by assuming containment measures remain constant for a period of 30 days.

**Results:**

Our analysis revealed that in the five European countries we compared, the use of different approaches definitively affected the effectiveness of containment measures for the Covid-19 pandemic.

**Conclusion:**

The evidence found in our research can be useful to inform policy makers’ decisions when deciding to introduce or relax containment measures and their timing, both during the current pandemic or in addressing possible future health crises.

**Supplementary Information:**

The online version contains supplementary material available at 10.1186/s12889-022-14088-7.

## Highlights

1. Different Covid-19 containment measures were adopted comparing five European countries

2. The earlier the restrictions were assumed, the better the response was achieved

3. Targeted measures before lockdown improved the curves compared with no restrictions

**Supplementary Information:**

The online version contains supplementary material available at 10.1186/s12889-022-14088-7.

## Introduction

Although the availability of vaccines to fight against SARS-CoV-2, various countries are still facing numerous barriers, some of them in paradox, such as vaccine hesitancy [1]. In fact, the progress of modern medicine has allowed the development in record time of effective and safe vaccines to prevent Covid-19 symptomatic infection, severe disease, admissions to hospital, and deaths [2–4]. Unfortunately, it has not been possible yet to ensure equitable access to vaccination throughout the world because there is not adequate availability of vaccines. Thus, it is essential to maintain non-pharmaceutical interventions to prevent transmission and reduce infections (https://www.who.int/emergencies/diseases/novel-coronavirus-2019/covid-19-vaccines). Actually, around the world, countries are still seeking to limit the impact of Covid-19 on health and society after more than 2 years of fighting SARS-CoV-2. To do this, governments are working as fast and fairly as possible to avoid new waves of the pandemic, minimize lives lost and maximize health, economic and social outcomes. Now, in 2022, we are more capable to identify new outbreaks early through timely and constant monitoring, to take appropriate measures to control the spreading of the virus (https://www.gov.uk/guidance/governments-approach-to-managing-local-coronavirus-outbreaks). We have a tool to control the virus, but large parts of the population for various reasons refuse to accept it. Countries have put in place legislative plans to address this issue. The concept, previously unknown, of a Covid Certificate has been introduced, in its various denominations and with the various peculiarities in different nations or geographical areas, showing also in this field differences at the international level [5].

Despite the availability of a winning tool - vaccines - due to the problems mentioned above, non-pharmaceutical interventions remain essential. They must be combined with a very accurate contact tracing and mass vaccination campaigns to be extended to as many countries as possible.

Through our research, we seek to understand how different mitigation and containment strategies have been important in the past, focusing specifically on the investigation of the early stages of the pandemic, so that we can emphasize how key they are today and for the future at minimizing the risk of potential additional waves of the Covid-19 pandemic.

**Fig. 1:**
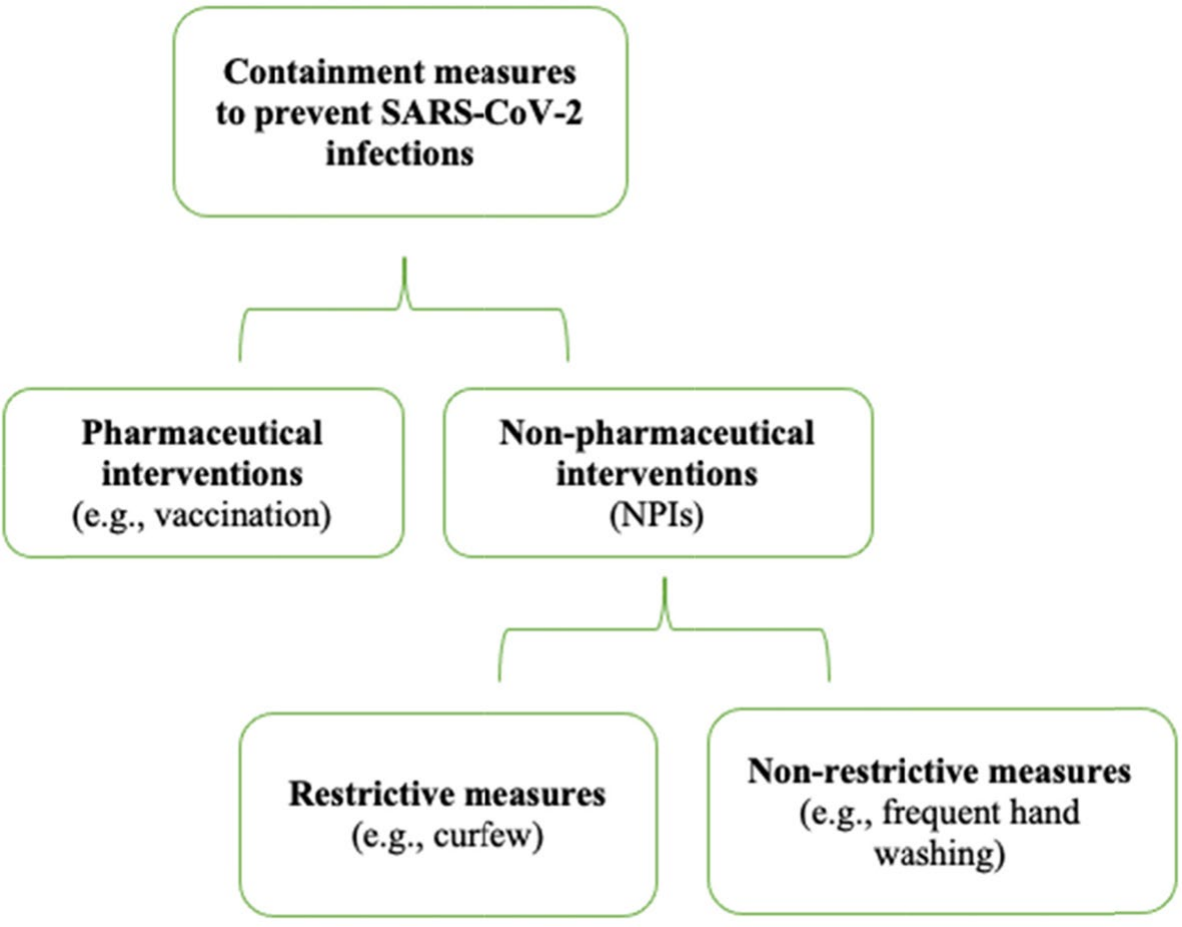
**Containment measures** - Classification of the containment measures adopted to prevent SARS-CoV-2 infections

The role of non-pharmaceutical interventions (NPIs) [6] in containing and mitigating contact rates in the population and thereby reducing transmission of the virus was assessed in this study [7]. Figure 1 provides a schematic representation of the containment measures adopted in order to prevent SARS-CoV-2 infections.

Non-pharmaceutical public health measures and their effects on person-to-person transmission at the individual level (physical distancing, use of face masks, wearing of eye protection) have been studied by the Covid-19 Systematic Urgent Review Group Effort (SURGE). The findings of this systematic review and meta-analysis support physical distancing of one meter or more and highlight the importance of the use of masks, respirators and eye protection in order to avoid or minimize the transmission of SARS-CoV-2 and its variants [8].

Stay-at-home orders, curfews and lockdown measures commonly occurred in countries with more than 10% daily increases in new cases [9] and the enforcement of strict physical distancing, along with robust level of testing, contact-tracing and household quarantine, contributed to keeping the disease at a level that did not exceed the capacity of a country’s health care system [10].

However, policy responses to Covid-19 seem to remain complex, context-specific and are rapidly evolving as different countries pursue varying approaches to manage the pandemic. Analyzing these policies can help to understand and assess government preparedness, timing of reactions and resilience (https://www.health.org.uk/news-and-comment/charts-and-infographics/covid-19-policy-tracker), [11].

Considerable efforts have gone into tracking health systems’ or governments’ responses to the pandemic, e.g., as part of the Covid-19 Health Systems Response Monitor. For example, Li et al. and Flaxman et al. conducted interesting comparisons of the approaches adopted by different countries in dealing with the pandemic [12]. In addition, previous research has shown that countries with less stringent lockdown measures have seen more pronounced surges (https://www.bsg.ox.ac.uk/).

This research aims to describe evidence on the effect of containment measures throughout the course of the pandemic in five European countries so far. “Containment measures” refer to the non-pharmaceutical and restrictive interventions. More specifically, the paper’s objectives included: 1) to compare the strength of containment measures adopted in the included countries; 2) to assess the timing of the introduction and relaxation of containment measures in relationship to the changes in the number of cases in these countries; and 3) to speculate about the effect the timing of relaxation measures had on the future course of the pandemic.

We chose France, Germany, Italy, Spain and the United Kingdom for our analysis because they were the first European countries to be hit by the pandemic (by end of February 2020, according to John Hopkins University information (https://github.com/CSSEGISandData/COVID-19). The results of this study may help decision makers better understand the effects of containment measures and the potential implications of lifting these measures too early. In fact, after studying the examples provided by the five countries and making statistical forecasts, we intend to provide a model that can help policy makers in making their important choices to address the current new waves of the pandemic and variants of the SARS-CoV-2.

## Main text

### Material and methods

The approach adopted by this study consisted of three steps. First, we built a “Containment Index” (C.I.) which considers nine parameters selected in order to make assessments on the strength of restrictive measures adopted by the five included countries. Second, dynamic epidemiological models were developed for forecasting purposes. Third, predicted case numbers, assuming containment measures remain constant, were compared to observed numbers for a period of 30 days following the relaxation of containment measures in each country.

#### Measuring intervention with the containment index

In order to measure government reactions to the pandemic, we followed a similar approach as the Oxford Covid-19 Government Response Tracker (OxCGRT) [13], but focused on containment measures only and tailored it to the countries in scope for our analysis (France, Germany, Italy, Spain and the UK).

We identified a set of nine parameters C_1,_ C_2_ ...C_9_ (only partially overlapping with OxCGRT), as described in Table 1, then scored and summarized them into a single summary index called “Containment Index” (C.I.).

**Table 1:**
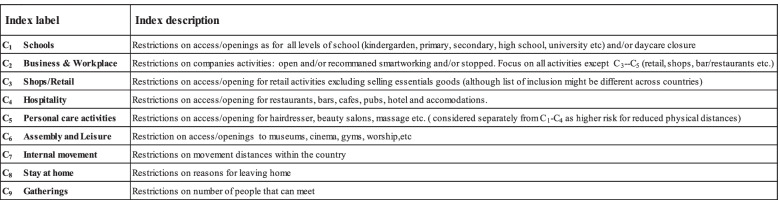
Containment Index - The nine parameters representing containment measures used to control the spread of the virus

Web research was conducted to collect the following information on governments’ responses in the five selected European countries: details of the actions they took, the start and end dates of those actions. Various bibliographic sources and reference sites were consulted for all countries analyzed in the study (https://www.euronews.com/2020/03/19/coronavirus-which-countries-are-under-lockdown-and-who-s-next), (https://en.unesco.org/covid19/educationresponse) to collect information on the restrictive measures respectively adopted in France (https://www.gouvernement.fr/en/coronavirus-covid-19), (https://www.connexionfrance.com/French-news/France-deconfinement-May-11-What-is-allowed-or-not-allowed-parks-beaches-schools-bars-restaurants-going-out-travel-transport), (https://www.thesun.co.uk/news/11391728/coronavirus-france-lockdown/), (https://www.france24.com/en/20200622-back-to-school-for-millions-in-france-as-more-covid-19-restrictions-lifted), (https://www.wsws.org/en/articles/2020/05/26/scho-m26.html), (https://www.bbc.co.uk/news/world-europe-52615733), Germany (https://www.bundesregierung.de/breg-en), (https://www.bbc.co.uk/news/world-europe-51999080), (https://kcrwberlin.com/2020/06/covid-19-in-berlin-and-germany-what-you-need-to-know/), (https://en.wikipedia.org/wiki/COVID-19_pandemic_in_Germany), (https://berlinspectator.com/2020/06/01/chronology-germany-and-the-coronavirus-3/), (https://www.euractiv.com/section/coronavirus/news/germany-extends-covid19-restrictions-but-announces-relaxation-of-some-measures/), (https://www.bavaria.by/information-coronavirus/), (https://www.thelocal.de/20200518/state-by-state-what-are-the-new-rules-for-eating-out-around-germany), (https://www.theguardian.com/world/2020/apr/28/germans-urged-to-stay-home-amid-covid-19-infection-rate-fears), (https://www.dw.com/en/what-are-germanys-new-coronavirus-social-distancing-rules/a-52881742), Italy (http://www.governo.it/it/coronavirus-misure-del-governo), Spain (https://www.spainenglish.com/2020/05/31/lifting-lockdown-spain-full-details-phases/), (https://www.rtve.es/noticias/20200605/mapa-desescalada-espana-fase-esta-tu-provincia/2013477.shtml), (https://www.boe.es/biblioteca_juridica/codigos/codigo.php?id=363&modo=2&nota=0&tab=2) and in the UK (https://www.health.org.uk/news-and-comment/charts-and-infographics/covid-19-policy-tracker), (https://www.gov.uk/government/publications/our-plan-to-rebuild-the-uk-governments-covid-19-recovery-strategy/our-plan-to-rebuild-the-uk-governments-covid-19-recovery-strategy), (https://www.instituteforgovernment.org.uk/explainers/coronavirus-and-devolution), (https://www.gov.uk/government/publications/actions-for-schools-during-the-coronavirus-outbreak/guidance-for-full-opening-schools), (supplementary material S0).

For each parameter, responses have been scored according to the below schema (Table 2). Scores are scaled to the same range from 0 (no restrictions) to 1 (max containment) in order to make them comparable. For some measures it was not possible to define an intermediate grading and we described it in the table as ‘*not assigned’*.

**Table 2:**
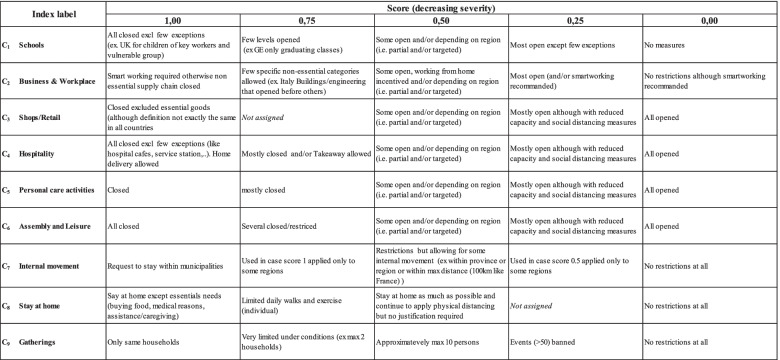
Containment Index score - The nine parameters can take a value ranging from 1 (maximum severity) to 0 (no restrictive measures): C_1_ = schools; C_2_ = businesses and workplaces; C_3_ = shops or retail; C_4_ = hospitality; C_5_ = personal care activities; C_6_ = assembly and leisure; C_7_ = internal movement; C_8_ = stay at home; C_9_ = gatherings

We tracked the start and end dates for each progressive restriction and then, for the data analyses and representation, the temporal axis was converted from days to weeks (Mon. - Sun.), following the rule that, for a given parameter, a week is assigned the score that is valid for the most (≥ 4) days in the week. In our analyses we tracked and analyzed data from the week starting on February 24th, 2020, to the week starting June 1st, 2020.

Finally, for each country/week, an overall Containment Index (C.I.) score was assigned. This is defined as the sum of the scores for the nine metrics, reflecting our assumption that each parameter evenly contributes to the overall index.

#### Modeling Covid-19 infection

We developed a Covid-19 Active Cases Curve Simulator which allows for daily predictions of “total active cases” based on the level and trajectory of active cases by country, region or state (public data from national and regional health commissions for the five countries analyzed: France [14, 15], Germany [16], Italy (https://github.com/pcm-dpc/COVID-19), Spain [17, 18] and the UK [19, 20], (https://coronavirus.data.gov.uk/#category=nations&map=rate).

The Covid-19 Active Cases Curve Simulator fits an upgraded version of the traditional Susceptible-Exposed-Infected-Removed (SEIR) compartmental models to allow for parameter customization by country, region or state and introduces dynamic contact rates to better predict the peak time and general trend. SEIR [21–26] is a well-known class of models that is widely used for modeling epidemic evolution and generating related predictions. The SEIR model, its general parameters and the other parameters introduced to adjust the model to better fit different nations or regions are explained in detail in in supplementary materials (Supplementary material S1).

The input to the models were confirmed Covid-19 cases (per PCR testing) that have been progressively made available by national/regional health commissions [10, 27], (https://www.ecdc.europa.eu/en/publications-data/download-todays-data-geographic-distribution-).

In some cases, public data have been amended and/or integrated after. This is not reflected in our analyses. In case of missing reported total cases for a few specific days, linear interpolation was applied.

#### Comparing the containment index across countries

Given the lack of an internationally accepted definition of terms, such as “lockdown” or “Phase Two”, we decided to define key milestones (and consequently phases) based on the evolution of the Containment Index over time to facilitate comparability across countries:

- Targeted period starts when the Containment Index becomes greater than 0 and if the first government measures are targeted to specific areas, regions or subsets of the population (called T0).
- Lockdown starts when the Containment Index becomes greater than 7 (the countries during lockdown can reach the maximum value of Containment Index, which is 9) (called T1).
- Phase Two starts when, after the peak, the Containment Index becomes lower again or equal to 6 (called T2).

Subsequently, analyses focused on comparing countries on a like-for-like basis, somewhat ignoring calendar time. Comparisons have been carried out in terms of descriptive analyses of the collected information (total cases and containment measures) as well as leveraging the predictions generated by the SEIR models implemented.

For each country, the Covid-19 Active Cases Curve Simulator has provided daily forecasts for total cases up to a maximum of + 150 days. For example, on March 1st, the Covid-19 Active Cases Curve Simulator generated predictions for total cases for March 2nd (+ 1 day) up to July 28th (+ 150 days) leveraging observed total cases up to March 1st; on March 2nd it generated predictions for March 3rd (+ 1 day) up to July 29th (+ 150 days) leveraging observed total cases up to March 2nd and so on. Day-after-day models progressively learn by incremental information made available which is reflected in more accurate predictions.

In the initial phase of the pandemic, one of the interesting objectives was to predict time to peak. But, as time passed, forecasts generated in previous months had been progressively overridden by actual data. Now, looking backward, we can leverage predictions made at specific points in time, compare different trajectories and correlate them to the different underlying restriction policies. In particular, based on predictions taken at Phase Two’s start (T2), we might speculate on what, under modelling assumptions, might have happened if lockdown restrictions continued for a longer time.

For such an exercise, we decided to focus on predictions at + 30 days. On one hand, we know that the accuracy of long-term predictions rapidly decreases given the extremely complex, fast-evolving factors that affect pandemic evolution (government restrictions as well as population compliance, mobility, individual behavior, social organization, climate, etc.). On the other hand, it is important to consider that potential effects of a containment measure might need approximately 14 days, according to the average duration for incubation based on clinical evidence and the recent studies confirming there is a lag for impacts of measures to be visible [28]. So, we need a forecast window reasonably greater than 2 weeks. Balancing the two considerations we chose a maximum timeframe of + 30 days.

## Results

France, Germany, Italy, Spain and the UK applied restrictive measures to contain the pandemic, but the timing and effect on the national curves of the Covid-19 cases varied. The graphical representation of these results is available in supplementary materials (Supplementary material S2).

### Initial reactions

Italy was the first western country to face the pandemic; the country had to react quickly and did not give up. During the week beginning on February 24th, some restrictive measures were applied only at a local level with a Containment Index of 4.5 (all confined locally to the so-called “red areas”). One week later, these measures were intensified and some of them were extended to the national level, while other restrictions were only adopted locally.

In Germany, the period of restricted measures that were partially applied at the regional level lasted for 4 weeks, twice as long as in Italy. It began, as in Italy, on February 24th, but in a very mild way, through the partial closure of schools and moreover only at a local level (C.I. = 0.25).

In Spain, the targeted local measures lasted only 1 week, starting on March 9th and concerned the partial local closure of some schools (C_1_ = 0.5).

In France, targeted restrictive measures were applied only on the first parameter: partial and local closure of schools from March 2nd to March 15th (C_1_ = 0.5).

The United Kingdom has having a particular attitude in dealing with the Covid-19 pandemic. Initially, they decided not to adopt any restrictive measures and recently as early as January 27, 2022 have lifted the requirement for masks and green passes. “In England, face coverings are no longer required by law. The government suggests that you continue to wear a face covering in crowded and enclosed spaces where you may come into contact with other people you do not normally meet” (https://www.gov.uk/government/publications/face-coverings-when-to-wear-one-and-how-to-make-your-own/face-coverings-when-to-wear-one-and-how-to-make-your-own).

### Country lockdowns

On March 9th, Italy was the first European country to enter a national lockdown and it lasted for 10 weeks, until May 3rd, with a C.I. = 9 and for the next 2 weeks until May 17th with a C.I. = 6.75.

From March 16th to May 10th, France was in lockdown for 8 weeks (C.I. = 9).

On March 16th, also Spain entered lockdown for 9 weeks. We observed C.I. = 9 only for 3 weeks (from March 30th to April 19th), after which it progressively decreased. Starting on May 11th, the last week of lockdown, closures were regional for all the analyzed parameters, leading to an overall C.I. = 7.

In Germany, during the lockdown that started on March 23rd, the value of the C.I. varied from 7.25 in the first 4 weeks to 6.75 in the two remaining weeks of the entire period (6 weeks).

On the same day as Germany, the UK government decided to enter a national lockdown that lasted 10 weeks (one of the longest lockdowns among the countries analyzed). During the first 7 weeks, C.I. = 9 and then, from May 11th to May 31st, C.I. = 7.75

### Entering phase two

The first country to enter the Phase Two was Germany: beginning on May 4th^,^ shops with less than 800 square meters and limited in-person shops could re-open, but with restrictions. From May 4th to May 18th, the C.I. was equal to 3.5. nationally then C.I. decreased to 3.25 on May 25th.

After entering Phase Two on May 11th, France maintained an overall C.I. = 5 for the remaining duration of the analyzed period. It was possible to move within a maximum distance of 100 km and schools gradually reopened at the beginning of Phase Two but with some restrictions: e.g., middle schools remained closed in red zones and gradually reopened in departments only in green zones.

On May 18th, Phase Two began in Italy at the national level: the C.I. achieved during the first 2 weeks of this phase was 3.25 (schools continue to be closed and there was no longer the stay-at-home requirement). During the week beginning on June 1st, the C.I. lowered to 2.5 due to no more restrictions on domestic movement.

On the same day as in Italy, Spain entered Phase Two and the restrictive measures were relaxed at the regional level with a C.I. = 5.25. From May 25th to the end of our analysis period, Spain achieved a C.I. = 4.

On June 1st in the UK, there was a gradual relaxation of the restrictive measures and the country entered Phase Two, but the level of attention and alertness was kept high, as reflected by a C.I. = 6, with a partial opening of schools and restrictions on gatherings

### Predictions about lockdown end and phase two start: (T2) + 30 days

The predictions made with our forecasting model focused especially on Lockdown end and Phase Two start. T2 + 30 would have been June 3rd in Germany, June 10th in France, June 17th in Italy and Spain and July 1^st^in the UK.

**Fig. 2:**
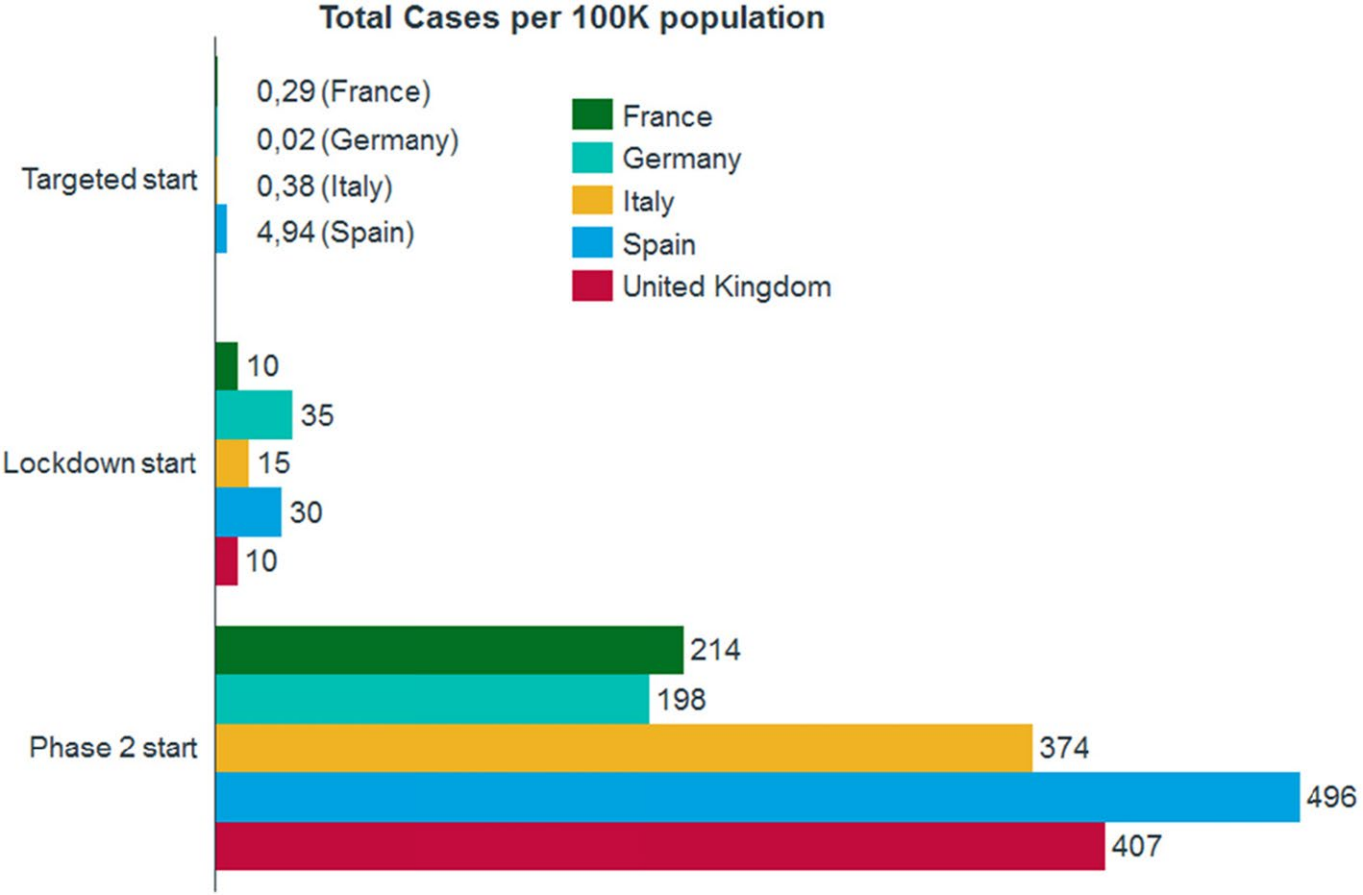
Total cases comparison for 100K inhabitants. At the beginning of initial targeted period, Spain recorded 4,9 cases per 100,000 inhabitants. When lockdown started, Germany and Spain recorded more than 30 cases per 100,000 inhabitants. At the beginning of Phase Two, all countries registered more than 190 cases per 100,000 inhabitants and Spain holds the record for this metric, having recorded almost 500 cases per 100,000 inhabitants

In Fig. 2, we analyzed the total number of reported positive cases per 100,000 of population in the five countries (https://www.health.org.uk/news-and-comment/charts-and-infographics/covid-19-policy-tracker), (https://www.spainenglish.com/2020/05/31/lifting-lockdown-spain-full-details-phases/), (https://www.rtve.es/noticias/20200605/mapa-desescalada-espana-fase-esta-tu-provincia/2013477.shtml), (https://www.boe.es/biblioteca_juridica/codigos/codigo.php?id=363&modo=2&nota=0&tab=2), (https://www.gov.uk/government/publications/our-plan-to-rebuild-the-uk-governments-covid-19-recovery-strategy/our-plan-to-rebuild-the-uk-governments-covid-19-recovery-strategy), (https://www.instituteforgovernment.org.uk/explainers/coronavirus-and-devolution), (https://www.gov.uk/government/publications/actions-for-schools-during-the-coronavirus-outbreak/guidance-for-full-opening-schools), [14–16, 27].

Considering the duration of the restrictive measures in the lockdown phase of the pandemic, Germany adopted the longest local (targeted) restrictive measures (4 weeks) and the shortest lockdown (6 weeks), which started later (when there were 35 cases per 100 K) compared to the other countries. Almost all the selected countries adopted restrictive measures for a period of 10 weeks while in Italy they took a period of 12 weeks (10 of which as lockdown). All the selected countries implemented a gradual closure of services and a progressive limitation of individual freedom except for the UK, since they didn’t adopt targeted measures.

**Fig. 3:**
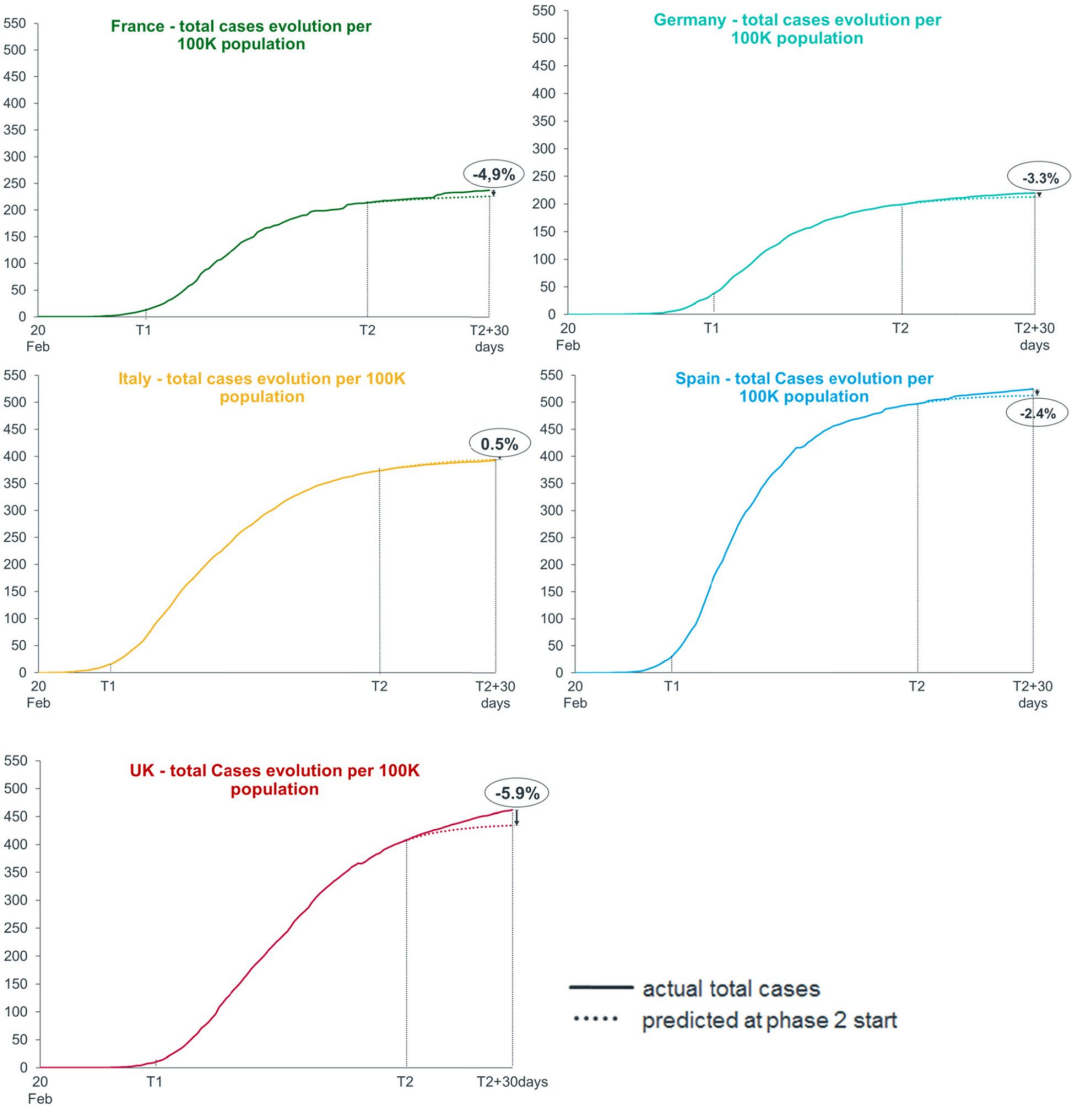
Epidemic curve. The figure shows curves representing the trend of actual total cases (continuous line) and curves representing the prediction taken at Phase Two start (dotted line)

In Fig. 3, we plotted the actual cases curve (continuous line) against predictions taken at T2 (dotted line) for each country (i.e., assuming lockdown containment measures were not eased). For instance, in France, Germany, Spain, and the UK, predictions under the assumption of additional lockdown weeks would have led to significantly lower cases (− 4.9, − 3.3%, − 2.4%, and − 5.9%, respectively at T2 + 30 days). However, for Italy, the real cases curve is very close to the predicted cases (+ 0.5% at T2 + 30 days), indicating that, had they maintained restrictions, they would not have changed the evolution on the curve.

**Fig. 4:**
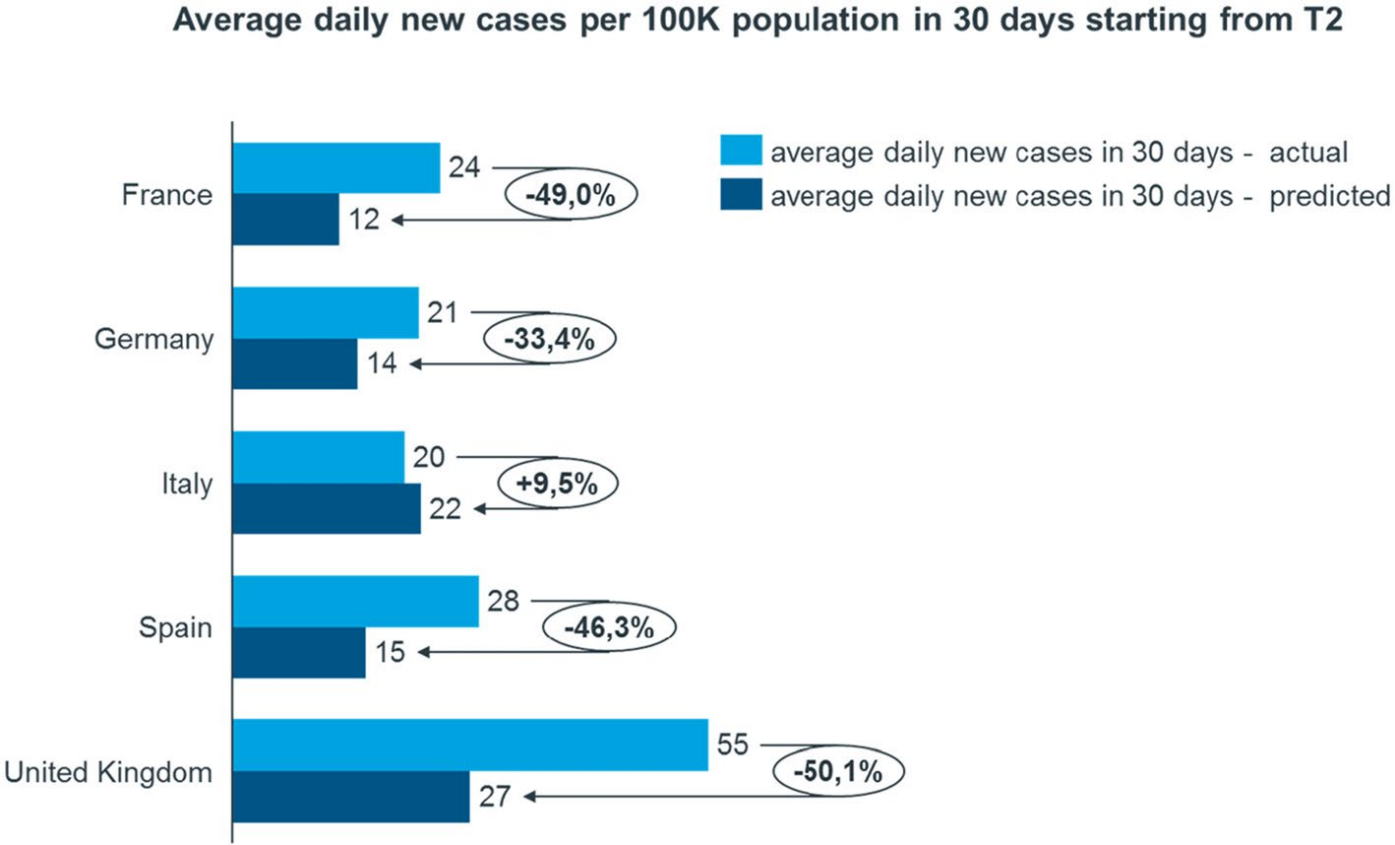
Average daily new cases. The figure shows the actual average daily new cases and the predicted ones in 30 days starting from Phase Two start (T2)

In addition, in Fig. 4 we have compared actual against predictions taken at T2 focusing on average new daily cases in the following 30 days. Here we can see that, according to predictions from our model, all countries except Italy might have benefited from a prolonged lockdown.

## Discussion

To face the Covid-19 pandemic, most European countries, and in particular those analyzed in this study (France, Germany, Italy, Spain and UK), have adopted diverse non-pharmaceutical and restrictive interventions ranging from case quarantine and isolation of contacts to the lockdown of entire populations. Assessing the impact of lockdowns and identifying the optimal strategies to manage the health crisis beyond lockdowns is of critical importance. To avoid the healthcare facilities overload, the severity of clinical cases and many deaths, it is has proved necessary to plan the response, adopting measures such as case finding, contact tracing, isolations and lockdowns of nations [29] and in parallel the large-scale vaccination campaign. Although these restrictive measures are crucial in minimizing the transmission of the SARS-CoV-2, the risk of pandemic resurgence when restrictions are relaxed and societies go back to a “business as usual” lifestyle has remained high. It is important to find a compromise between public health requirement and the economic and social needs of the population, analyzing the different restrictive measures in order to improve these measures aimed at reducing the spread of SARS-CoV-2 [10].

The Oxford Covid-19 Government Response Tracker (OxCGRT) constantly collects information on measures taken by different governments to contain the effects of the pandemic and now has data from more than 180 countries. Surely this is an important tool for worldwide comparisons but, in our study, we thought it would be helpful to understand these comparisons across a limited number of European countries. As a result, we went for a tailored definition of all parameters for Containment Index definition.

Other studies have addressed the topic of quantifying the effect of containment actions by looking at a rather high number of countries: Talic S et al. performed a meta-analysis to research the effect of handwashing, mask wearing and physical distancing measures on incidence of Covid-19. They concluded that these several personal and social protective measures are associated with decrease in virus circulation [30].

Iezadi S et al. in their meta-analysis showed that NPIs has determined a decrease in the COVID-19 reproduction number, daily case growth rates, daily death growth rates and COVID-19 daily ICU admission. It emerged that early enaction of lockdown, which predates the explosion in the number of positive cases, has been followed by a shorter duration of the lockdown itself and a smaller increase in the rate of case growth in the period after the application of this maximum containment measure [31].

Li et al. conducted a modelling study across 131 countries [28] and Flaxman et al. studied the effect of major interventions across 11 European countries [12]. Conversely, our analyses aimed to describe, in a comparative way, different containment approaches implemented by the five countries and to qualitatively correlate them with the evolution of the pandemic. Our analyses were aimed at assessing whether mild measures or delays in adopting the different types of restrictive measures could have influenced the number of Covid-19 cases and the evolution of the epidemic curve, in order to be able to guide the near-future choices of governments, policy makers and health authorities in the best possible way. Consequently, based on the evolution of case numbers and the adopted restrictive measures in Europe, it is possible to make the following considerations.

Following the diagnosis of the first patient affected by Covid-19 in Germany on January 27th, Germany implemented much milder and shorter restrictive measures in comparison to the other four countries. All restrictions were applied to every German state and were kept in place for a total of 6 weeks but there was never a total closure and, unlike the other European countries, Germany never issued a curfew. It should be noted that during the lockdown Germany never adopted the stay-at-home requirements in an absolute way (C_8_ = 0.5). Germany would have had a reduction of new cases from an extension of the lockdown and likely from the application of more restrictive measures as well, given the trend of the T2 + 30 curve in our predictive model (− 3.3%).

As early as January 30th, the Italian government ordered the blocking of air traffic from China, the first European government to do so. This was the beginning of the application of a long list of restrictive measures in Italy. After the first 2 weeks of targeted measures, Italy adopted one of the longest periods of lockdown (10 weeks, like in the UK) compared to the other selected countries. The adoption of such stringent measures has made a unique and indispensable contribution to the ongoing fight against the pandemic. In fact, as we observed in our predictive model, it seems that the relaxation of the restrictive measures was introduced at the right time because there would have been no net reduction of daily cases if the country would have entered Phase Two later (T2 + 30 = 0,5%). This shows a big difference between Italy and the other four countries analyzed in our study regarding the entry of the countries to Phase Two.

As France experienced the first wave of the Covid-19 pandemic, a nationwide lockdown was implemented to curb the dramatic increase in the number of patients in critical conditions. The country is the only one to have maintained a total closure for all parameters (C.I. = 9) during the entire lockdown. However, this seems not to have been enough. In fact, considering our predictive model, France could have benefited from an extension of the lockdown duration, being able to obtain a further reduction in the number of new cases (T2 + 30 = − 4,9%).

Following the story of Italy, Germany and France, Spain adopted a long lockdown (9 weeks) but maintained the maximum level of restriction for the shortest time (the maximum C.I. = 9 was true for only 3 weeks, compared to Germany where the maximum C.I. = 7.25 was held for 4 weeks). Spain is also the only country to have applied all restrictive measures at the targeted level before Phase Two (i.e., during the lockdown). Both these attitudes could justify the trend of the T2 + 30 curve of our preventive model (− 2,4%). The country probably should have applied more stringent restrictive measures and should have delayed the application of restrictive measures at the regional level, considering that Spain is the country that entered Phase Two with the highest number of cases per 100 K population (496 total cases per 100 K population) among the five countries analyzed.

The UK, despite not having gone through a targeted period, has applied very stringent restrictive measures during the lockdown and the country has very gradually entered Phase Two. The UK lockdown lasted for the same duration as the Italian lockdown, but the country started to adopt the first restrictive measures 4 weeks later than Italy did (March 23rd in the UK compared to February 24th in Italy). In our preventive model, the country would have benefited greatly (− 5.9%) from a delayed entry into Phase Two. This can be justified, as in France, by a consistent circulation of the virus that was difficult to control, already existing at the beginning of the adoption of the restrictive measures in the country. The UK has decided not to take some measures on a regional basis, as has been done by France, Germany, Italy and Spain.

The easing of the measures was carried out in a more or less gradual and cautious way by all five countries: for Phase Two, Germany had reported the lowest number of total cases per 100 K population among the five countries of the study, followed by France, Italy, the UK, and finally Spain (which loosened with the majority of cases). We can say that, compared to the other four countries analyzed, Germany was the first country to adopt a relaxation of restrictive measures: Germany began its first steps to ease restrictions on April 20th (during lockdown). Schools were reopened in Germany (C_1_ = 0.75), and approximately 25% of the students were allowed to go to school. Schools were soon opened in France (on May 11th) but there was a maximum number of 15 students for each classroom (C_1_ = 0.5). During Phase Two in Spain and Italy, schools continued to be closed. Particular attention should be paid to the fact that when Germany entered Phase Two, it had not imposed any restrictions on internal movement of citizens. Further, Italy was the only country that renounced the stay-at-home requirements immediately when it entered the Phase Two.

Consistent evidence can be observed in our study and in others previously published on the impact of restrictions’ ease. In particular, our findings on restrictive measures’ effects are in line with the findings from Flaxman and colleagues [12] who assessed the impact of different NPIs among 11 European countries, but not their effects. Flaxman and colleagues reported clearly that several NPIs (e.g., school closure and public events ban) combined with lockdown had a large effect (81%) on reducing transmission. Our findings are also consistent with results from a 131 cross-country study by Li and colleagues [28]. They observed that individual NPIs are associated with a reduced SARS-CoV-2 transmission and that the effect of introducing and lifting NPIs is delayed by 1–3 weeks, with a longer delay occurring when NPIs are lifted: a resurgence in Covid-19 cases has been reported in some countries that lifted some of these NPIs.

Based on our observations, France and the UK had a small number of positive cases at the beginning of the pandemic and during lockdown, while Spain experienced high caseloads during the first wave of the pandemic and a constant increase of positive cases for SARS-CoV-2 during the second phase.

Further, looking at the evolution of the epidemic curves in the different countries, if we assume that lockdown containment measures were not eased (i.e., predictions until T2 + 30, the dotted line in the Fig. 3), we obtain interesting information when comparing countries. If the total blocking measures were prolonged, they would have led to a greater reduction of cases in the UK (− 5.9%), France (− 4.9%), Germany (− 3.3%) and Spain (− 2.4%), while Italy (+ 0.5%) would not have achieved substantial improvements (the zeroing target had already been achieved).

When speculating about possible reasons for the different effects in easing lockdown measures, we mainly observed different behaviors related to school measures. Schools remained closed in Phase Two in Italy and in Spain while the other three countries gradually reopened (C_1_ was 0.75 at the beginning of Phase Two in France, in Germany and in the UK). In Germany, only the last year of each school level restarted (approximately 25% of students). In France, kindergarten and primary school children returned to school on a voluntary basis, middle schools were gradually reopened in the green zones while they remained closed in red zones. Like France, the UK began easing restrictions on schools starting with the opening of kindergarten and primary school.

As for other parameters such as businesses and workplaces, shops and retail, hospitality, personal care activities, assembly and leisure, internal movement, stay at home and gatherings, only Italy adopted targeted restrictions before the lockdown and Germany only had partially adopted restrictions for shops and retail, hospitality, assembly and leisure, and gatherings. In Italy, the early partial - and then total - restrictions concerning all the mentioned parameters can justify the good result reached by the predictive model T2 + 30. Furthermore, based on the number of Covid-19 cases, Germany was the first to introduce targeted measures (at 0.02 total cases per 100 K population) and Spain the last one (at 4,94 total cases per 100 K population). The UK entered lockdown with the same number of cases as France (10 total cases per 100 K population), but the UK started applying the restrictive measures a week later than France and without previous targeted measures reporting a number of Covid-19 cases almost double than France at the beginning of Phase Two (407 cases per 100 K in the UK vs. 214 cases per 100 K in France).

The results of our model speculate the evolution of the pandemic curve if the containment measures were prolonged and suggest that all the countries except Italy would have benefited from longer restrictions at the time of Phase One, although it was not possible to clearly quantify the contribution different behaviors had. Overall, we can say that there were three main factors that affected the SARS-CoV-2 pandemic in the above mentioned European countries: 1) the time when containment measures were adopted according to the epidemic curve (the earlier were the better); 2) the duration of containment measure adoption (2 weeks of early restrictive measures before the lockdown gave better results than no restrictive measures before the lockdown); 3) the number of Covid-19 cases before easing containment measures (the fewer the better before concluding the lockdown). We agree with Iezadi S et al. that the NPIs has had successful impacts on containing the spread of SARS-CoV-2, despite the substantial impacts on economies and mental health. In addition to addressing issues regarding universal access to vaccines, considering the severe consequences of national lockdown and other restrictions, these interventions should be accompanied or mitigated by the adoption of other NPIs such as contact tracing, the use of face masks and suspected/patient case isolation strategies [31].

## Conclusions

Governmental policies were found to play a crucial role in delaying the Covid-19 infection early spread [32] and the Case fatality rate, CFR [33]. From a detailed analysis regarding the introduction of some restrictive measures (nine analyzed parameters) in the five countries of our study (France, Germany, Italy, Spain and the UK) and their subsequent lifting, their importance for the mitigation of the Covid-19 pandemic has emerged. In fact, the UK began to tackle SARS-CoV-2 with restrictive measures later than others and, despite having applied these measures for a long time, had greater difficulty in the fight against the virus. On the other hand, Italy, the first European country to be hit and to promptly implement the restrictive measures on all nine parameters analyzed, would not have benefited from a possible extension of the lockdown. In Germany, the restrictive measures were applied promptly but in a much milder way than in the other four countries; despite having the lowest number of cases (per 100 k population) when Phase Two started, the country would have possibly benefited from prolonged restrictive measures. France began rather promptly to adopt the restrictive measures against SARS-CoV-2 but the lockdown lasted only 8 weeks and the country would have benefited from a delayed entry into Phase Two. Spain applied restrictions limited to some regions during the lockdown. It was the country that entered Phase Two with the largest number of cases.

Reintroducing or easing restrictions are undoubtedly important decisions for governments because they have a relevant impact on the economy as well as on the psychological integrity and health of the population. For this reason, such decisions should be made by weighing many factors, including the epidemiological situation of the country, the capacity and resilience of the health system and the attitude and habits of the population. Our findings provide additional evidence that can inform policy makers’ decisions on introducing and relaxing of the different containment measures and their timing. Given the features of the Covid-19 pandemic, extensive case findings and isolations would be required to progressively lower the intensity of interventions and allow for the partial release of the socio-economic pressure and avoid the healthcare system exceeding saturation. We can only end the pandemic in one place by ending the pandemic everywhere. The entire world has the same goal: decrease Covid-19 cases. The SARS-CoV-2 countries’ response was very different: in some cases it was a failure, in others a success. Surely the most important lesson learned is that we can definitely defeat the invisible enemy and its emerging variants by improving our knowledge about the virus, its effects and how to contain both.

### Limitation of the study

Overall, we acknowledge challenges and limitations regarding our analyses and interpretation of Covid-19 dynamics. First of all, in the initial phase of the pandemic, overall detection and reporting of positive cases was less accurate than in the following periods, causing models to learn from a likely underestimated total cases curve [34, 35]. Second, at the beginning, the number of tests (swabs) was insufficient, so a great majority of asymptomatic subjects were not tested. Third, we are aware that there might be gaps in the data used, in some specific days or due to country-specific reporting systems. In case of missing information, we replaced it with linear interpolation between adjacent observations. Fourth, as for the different containment actions, our scoring system considers the start and end dates as well as the level of restriction declared by each country according to our investigation. The level of compliance from population is not considered as it was not possible to properly measure it.

We acknowledge also limitations of the SEIR method used in our analysis: the removed subjects (R) are the recovered and dead cases that are assumed to be immune to the disease and would not be reintroduced to the susceptible (S). Model performances (and predictions generated) highly depend on accuracy, transparency, and promptness of data used as inputs. Related to this, in some cases, countries implemented changes in the reporting and testing approach, which would potentially affect the reported dynamics for case evolution. As for the model, as expected, accuracy of predictions decreases along time and models implemented in the Covid-19 Active Cases Curve Simulator maximize short term (7-day) predictions. Also, it is important to underline that predictions at each step for each country are generated by the models under the assumptions that conditions are not varying. In our speculations, we have considered “what if” lockdown measure continued for some weeks more, but predictions cannot take into account additional external factors such as changes in populations’ behaviors (self-reducing contacts, limiting social activities, increased hygiene/disinfection in everyday life).

## Supplementary Information

**Additional file 1.**

**Additional file 2.**

## Data Availability

It was not possible nor needed to involve patients or the public in the design, conduct, or reporting of our research. Data were obtained from publicly available sources. All data generated or analysed during this study are included in this published article and its supplementary information files.
