# Supplementary material for: A cross-country comparison of Covid-19 containment measures and their effects on the epidemic curves
Source: BMC Public Health. 2022 Sep 17;22:1765. doi: 10.1186/s12889-022-14088-7 (PMC9482299; doi:10.1186/s12889-022-14088-7)
Supplement: Supplementary file 1 — Additional file 1. [file 12889_2022_14088_MOESM1_ESM.docx]

**A cross-country comparison of Covid-19 containment measures and their effects on the epidemic curves.**

**Supplementary material S1 - SEIR Model**

**Supplementary Figure S1** - **SEIR diagram:** S = people susceptible to the infection; E= people which have been exposed to the virus with a long incubation duration, but don’t show any symptom; I= people infected/confirmed cases; R= people removed from the susceptible group because they recovered or died. Further, $\beta$ is the infectious rate; $\alpha$, the incubation rate and γ is the recovery rate.

| **Parameters of SEIR model** | **Explanation of the parameters** |
| --- | --- |
| $\boldsymbol{\beta}$ | **Infectious rate** controls the rate of spread. Due to the unawareness of the public and virus variation, $\beta$ is supposed to increase in the early stages and decrease under strict control policies. For this reason, in our upgraded version of SEIR model, $\beta$ is assumed to be changing over time according to the following formula (70): $\beta\left( t \right)=\frac{Ce^{-a(t+b)}}{\left( {1+e}^{-a(t+b)} \right)^{2}}$ We suppose $\beta$ follows the slope of a logistic function where *a* is the regularization parameter, *b* is bias and *C* is a scaling constant. By training the history data, we will get an estimate of the above parameters and consequently an estimate for $\beta$, to use in the dynamic SEIR model for prediction every day. |
| $\boldsymbol{\alpha}$ | **Incubation rate** is the rate at which latent individuals become infectious, calculated as the inverse of the average incubation period. We use the average duration of incubation from SARS as a reference (7 days), so the incubation rate was assumed to be 1/7 (71, 72). |
| $\boldsymbol{\gamma}$ | **Recovery rate** is calculated as the inverse of recovery period. We assume the average recovery period is 14 days (73). |

**Supplementary Table 1.1 / Table S1.1** **Parameters of SEIR model**: β infectious rate, α incubation rate and γ recovery rate.

| **Parameters** | **Explanation of the parameters** |
| --- | --- |
| ***Mortality rate*** | It is calculated as the rate of death over total confirmed cases. |
| ***η*** | It is used to estimate the initial exposed (E). The SEIR model uses the last observation as the initial point. However, the exposed (E) is not available from public databases so we compute E by multiplying I with a scaling up parameter, *η*, which may be close to the actual R_0_ but is achieved by training the history data. |
| ***IR Rate*** | It can adjust the recovery rate at various stages to have more accurate predictions if the nation/region has a large number of emerging cases or limited medical resources. |
| ***AI Rate*** | It can adjust the incubation rate at various stages to have more accurate predictions if the nation/region has either enormous or limited testing/examination capacity. |

**Supplementary Table 1.2 / Table S1.2** **Other parameters added to the SEIR model**: mortality rate, η to estimate the initial exposed, IR Rate and AI Rate.

The following system of nonlinear ordinary differential equations describes the transmission of virus:

These hold true where S+E+I+R=N is the total population, and infectious rate, $\beta$, incubation rate, $\alpha,$ and recovery rate, $\gamma$ are greater than 0.

**Supplementary material S2**

In **Supplementary** **Figure S2** we created a graphic representation of the various parameters analyzed in our study, arranging the five countries in alphabetical order.


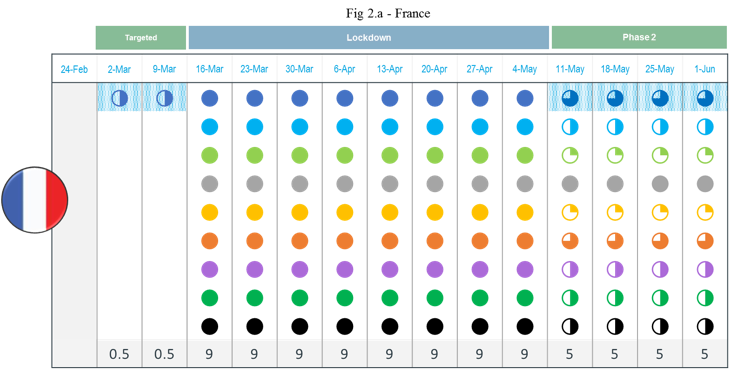

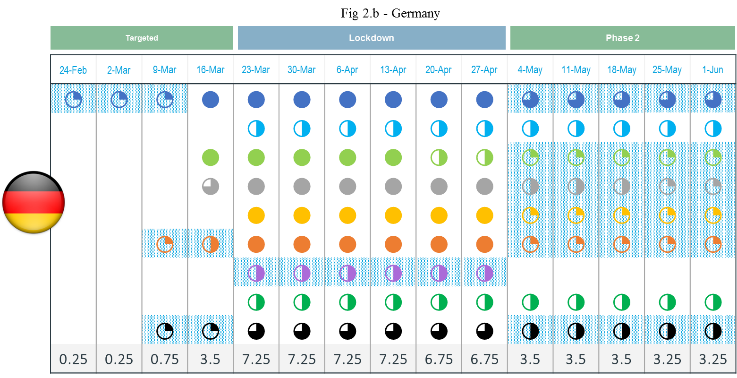


***
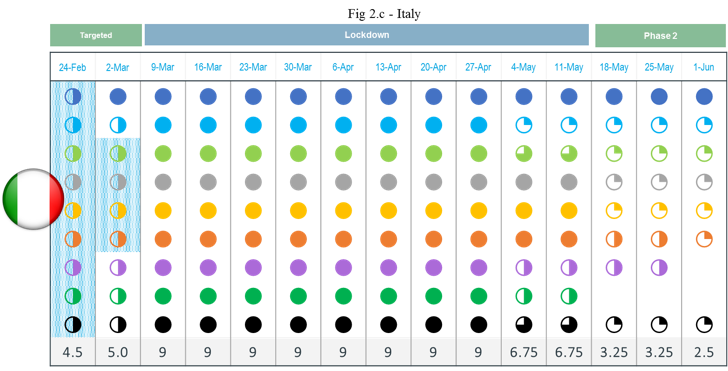

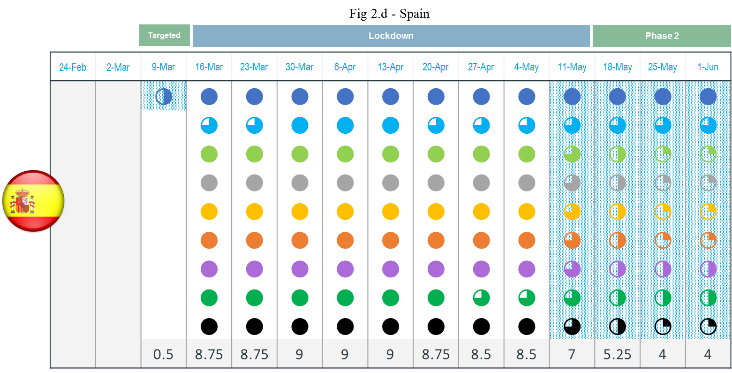
***


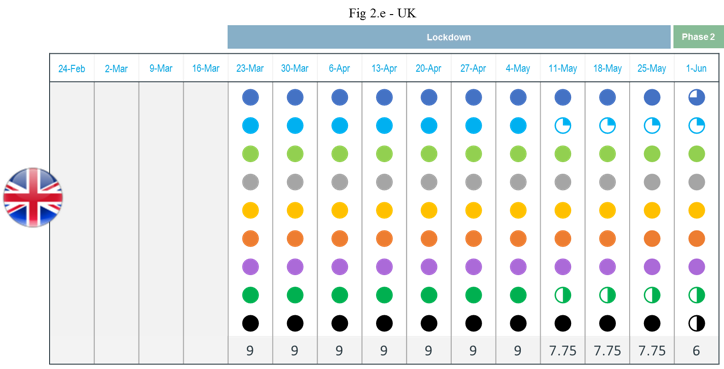

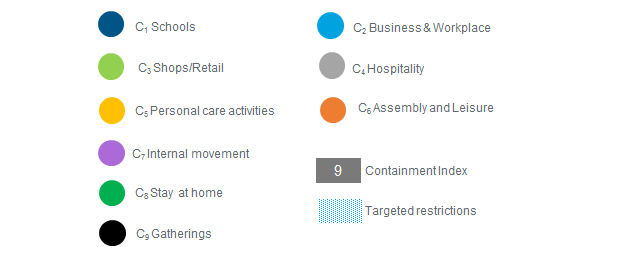


**Supplementary** **Figure S2.** Detailed analysis of the nine parameters used to measure the Containment Index in the studied five European countries from Feb 24^th^ to Jun 1^st^. Fig 3.a shows the values of the nine parameters during the period analyzed in France; Fig 3.b shows the values of the nine parameters during the period analyzed in Germany; Fig 3.c shows the values of the nine parameters during the period analyzed in Italy; Fig 3.d shows the values of the nine parameters during the period analyzed in Spain; Fig 3.e shows the values of the nine parameters during the period analyzed in the UK.
